# Dataset produced by automated sand-rammer, clay-gun, and plate viscometer for three different tap-hole clays

**DOI:** 10.1016/j.dib.2021.106819

**Published:** 2021-01-30

**Authors:** Joalet Dalene Steenkamp, Charlotte Lindstad, Lars Lindstad

**Affiliations:** a Mintek, Randburg 2125, South Africa; b University of the Witwatersrand, Johannesburg 0001, South Africa; c Elkem Carbon, Kristiansand 4621, Norway

**Keywords:** Tap-hole clay, Workability, Rheology, Clay-gun, Sand-rammer, Plate viscometer

## Abstract

In pyrometallurgical furnace operation, tap-hole clay is injected into the tap-hole using a clay-gun. The goals are to stop the metal and/or slag from flowing and to create a seal between the furnace contents and the environment. The rheological properties of the tap-hole clay play an important role in this process. Some commercial manufacturers of tap-hole clay report the workability index (WI) of their products, based on sand-rammer technology and standardised procedures. In the paper presented here, datasets are presented for three different tap-hole clays where the effect of the choice in clay on the pilot-scale clay-gun was demonstrated and an automated sand-rammer was utilised to determine the standard WI as well as an extended WI. A plate viscometer, utilised in the characterisation of electrode paste, was applied as potential alternative technology utilised when characterising the rheological properties of tap-hole clays. In all three instances, the data collection process was automated with raw and/or filtered data, available as Excel spreadsheets, published in an online repository. For the purpose of this paper, the data was analysed and presented as graphs or in tables. The data will be of future use for further studies into the effect of tap-hole clay rheology on clay-gun performance.

**Specifications Table**

**Table:** 

Subject	Continuum mechanics
Specific subject area	Characterisation of the rheological properties of two industrial and one experimental tap-hole clays applied when closing tap-holes of pyrometallurgical smelters
Type of data	Tables, graph
How data were acquired	Pilot-scale clay-gun (DDSA/O-M/MTK1160) Automated sand-rammer (R&D Carbon, RDC-194) Plate viscometer (Viscometer M11)
Data format	Raw, filtered, analysed
Parameters for data collection	For three different tap-hole clays, two industrial and one experimental: • Changes in hydraulic pressure applied to the piston used to push the clay out of the barrel of the clay-gun • Changes in sample height as a function of the number of rams for the automated sand-rammer • Measured force as a function of plate height at a fixed plate speed for the plate viscometer
Description of data collection	Three different tap-hole clays, two industrial and one experimental, were subjected to three different experimental techniques to determine their rheological properties. Experiments were conducted on the automated sand-rammer and plate viscometer in Kristiansand and on a pilot-scale clay-gun in South Africa. Room temperature conditions prevailed. During July 2018 the average temperature in Kristiansand was 20 °C [2] and in Johannesburg 10 °C [3]
Data source location	Mintek Johannesburg South Africa 26°05′19′′S 27°58′39′′E Elkem Carbon Kristiansand Norway 58°07′40′′N 7°58′04′′E
Data accessibility	Repository name: Dataset produced by automated sand-rammer, clay-gun, and plate viscometer for three different tap-hole clays Data identification number: https://doi.org/10.17632/ddrn6hpkdj.2 Direct URL to data: https://data.mendeley.com/datasets/ddrn6hpkdj/2

## Value of the Data

• The data produced here is a subsequent study on work done in 2017 on the characterisation of tap-hole clays which was presented at INFACON XV in 2018 [1]. All results presented here are new.
• The data produced is beneficial to furnace operators as it demonstrates the effect of choice in tap-hole clay on clay-gun performance. This could result in improved tap-hole clay selection and safer furnace operation.
• The data produced is beneficial to producers of tap-hole clays as it demonstrates the type of technologies available to characterise the rheological properties of tap-hole clays. This could result in improved tap-hole clay design and development.
• The data will be useful for further studies into the effect of tap-hole clay rheology on clay-gun performance, more specifically for the validation of mathematical models used to describe the effect of the rheological properties of the tap-hole clay on clay-gun behaviour. This could result in improved clay-gun design.

## 1 Data Description

**Fig. 1:**
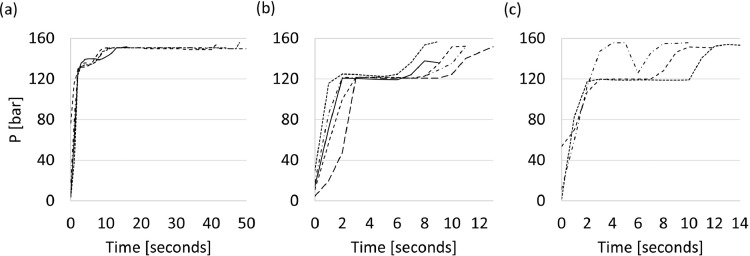
Plugging pressure, as measured in the hydraulic system, as a function of time for three different tap-hole clays: (a) Clay A, (b) Clay B, and (c) Clay C. Each graph represents datasets for 3–5 experiments.

Fig. 1 contains the plugging pressure, as measured in the hydraulic system of the clay-gun, as a function of time for the three different tap-hole clays. Each graph represents datasets for 3 to 5 experiments and the filtered data is available in the repository as a Microsoft Excel file named “Fig. 1”.

**Table 1:** Average workability index per type of clay calculated for five data points per clay.

	Clay A	Clay B	Clay C
Average WI_3_	0.5	12.5	19.1
StDev	0.1	4.8	2.0

**Fig. 2:**
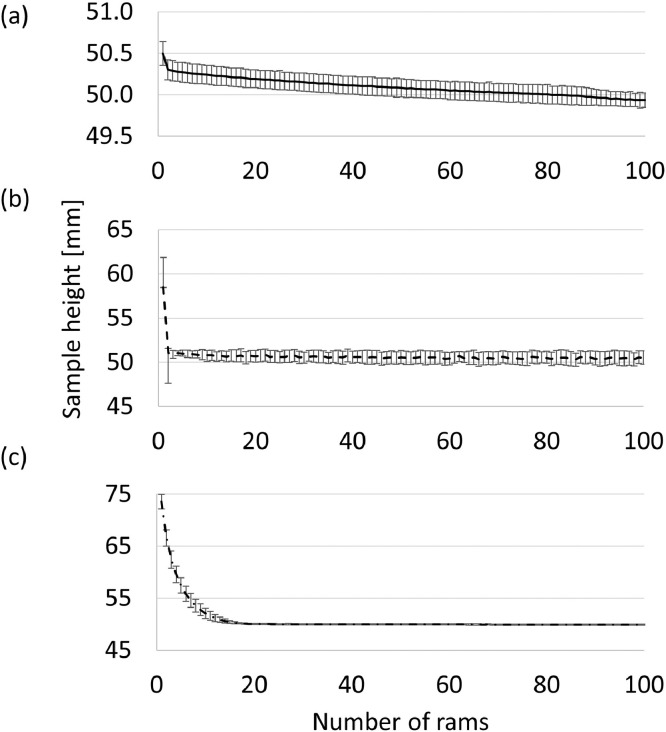
Average sample height per type of clay – calculated for 5 experiments – as a function of number of rams for (a) Clay A, (b) Clay B, and (c) Clay C. Error bars indicate standard deviation in measured height.

**Fig. 3:**
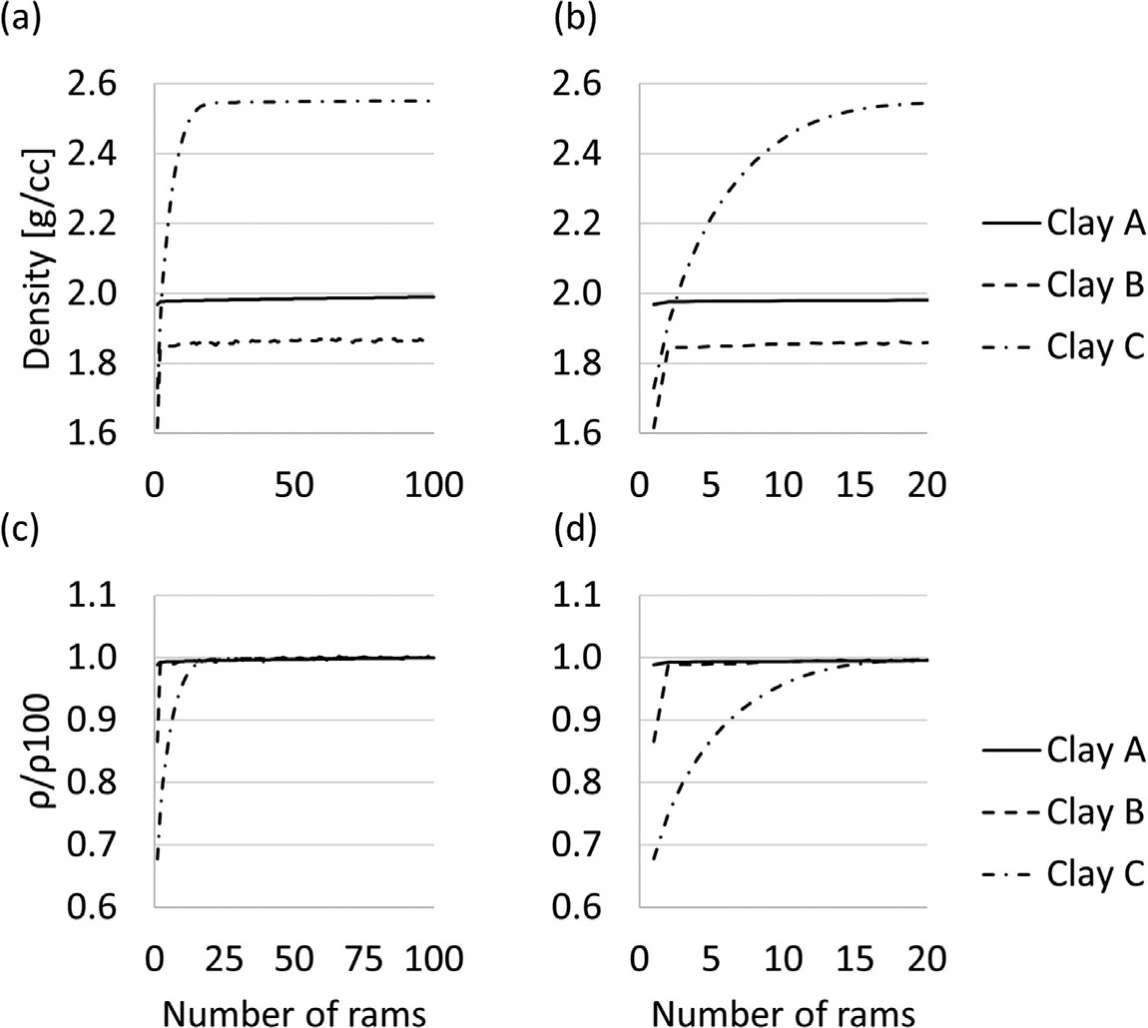
Average density per type of clay – calculated for 5 experiments – as a function of number of rams (a, b); and the ratio of density after a specific number of rams to density after 100 rams, per type of clay, as a function of number of rams.

**Fig. 4:**
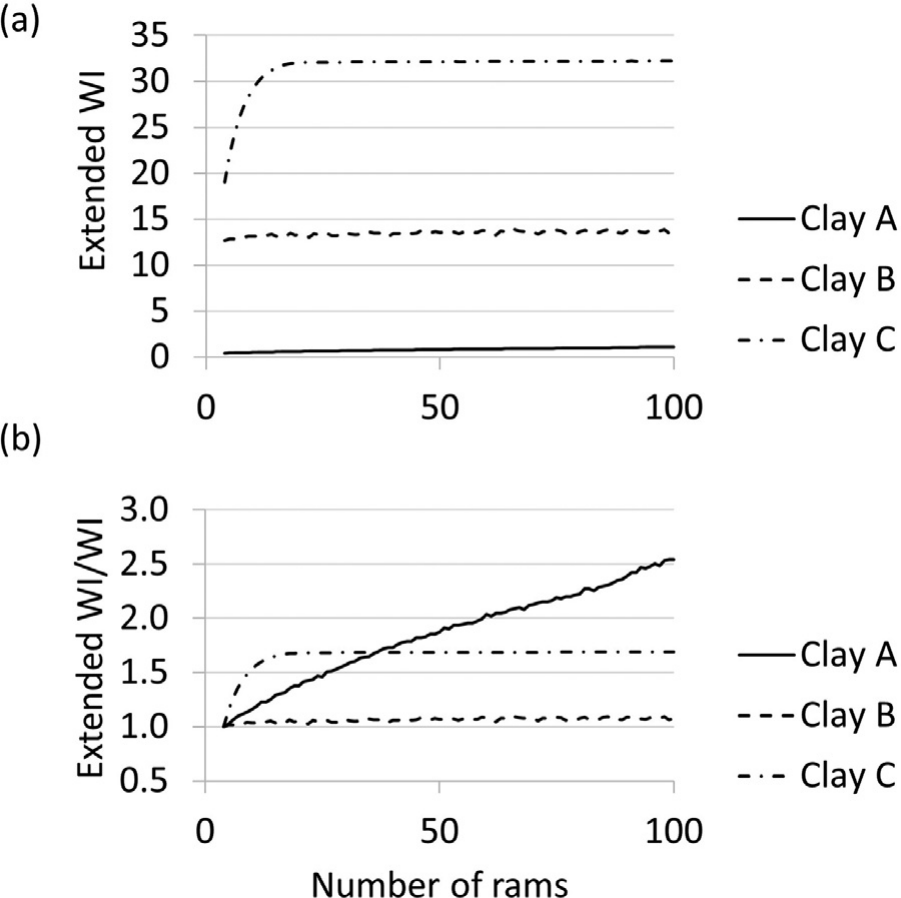
Extended workability index per type of clay calculated for results in Fig. 2(a); and the ratio of extended workability index to workability index for each type of clay.

In Table 1, the average workability index (WI) per type of clay, determined by the automated sand-rammer according to standard methods [3], [4], were summarized. The raw and analysed data sets are available in the repository as a Microsoft Excel file named “Table 1 Fig. 2 Fig. 3 Fig. 4”.

In Fig. 2, the effect of the number of rams on the sample height is reported for each of the different clays. The raw and analysed data sets are available in the repository as a Microsoft Excel file named “Table 1 Fig. 2 Fig. 3 Fig. 4”.

The results in Fig. 3, illustrates the effect of ramming on the density of the clay samples. The raw and analysed data sets are available in the repository as a Microsoft Excel file named “Table 1 Fig. 2 Fig. 3 Fig. 4”.

The results in Fig. 4, illustrates the effect of ramming on the extended workability index of the clay samples. The raw and analysed data sets are available in the repository as a Microsoft Excel file named “Table 1 Fig. 2 Fig. 3 Fig. 4”.

**Fig. 5:**
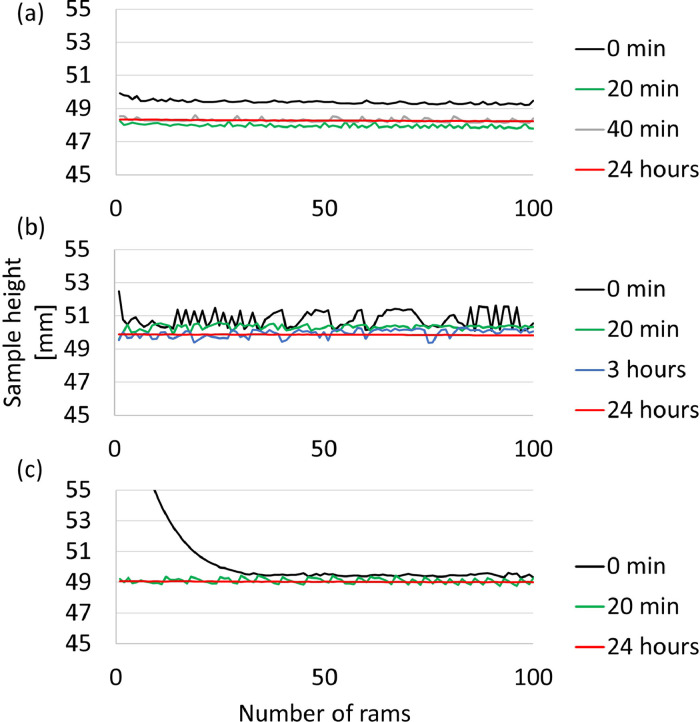
Sample height as a function of number of rams for (a) Clay A, (b) Clay B, and (c) Clay C for different periods of standing time.

Fig. 5 contains the sample height as a function of number of rams for the three different clays, for different periods of standing time (time from filling of the crucible for the sand-rammer until conducting the experiment) ranging from zero (initial tests) to 24 h. The raw and analysed data sets are available in the repository as a Microsoft Excel file named “Fig. 5”.

**Fig. 6:**
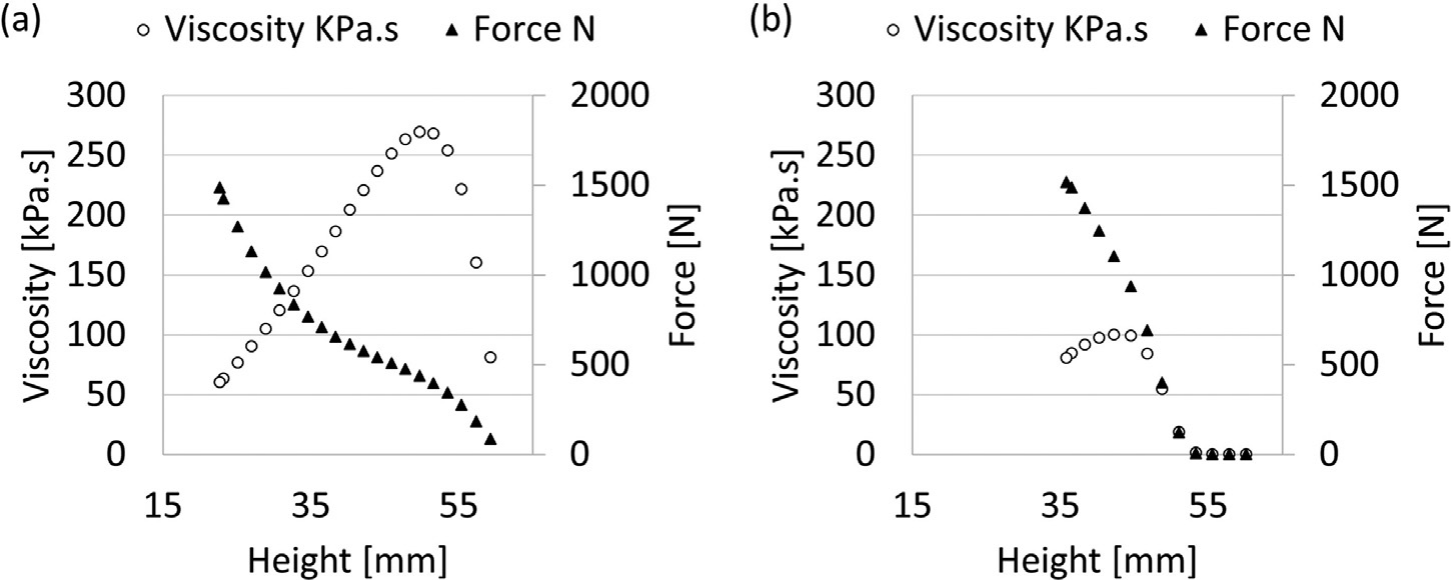
Measured force and calculated viscosity as a function of plate height for (a) Clay B, and (b) Clay C at a fixed plate speed of 450 mm/min. Results are for one run on fresh clay only.

**Fig. 7:**
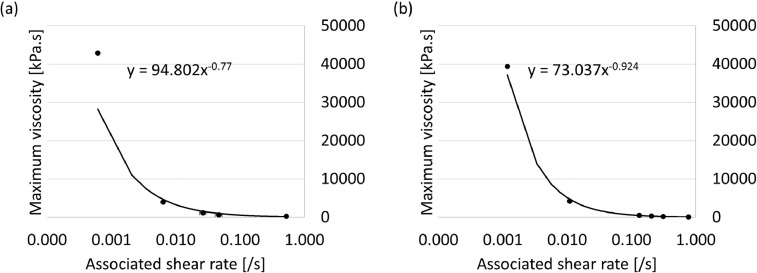
The linear relationship between maximum viscosity and associated shear rate determined for (a) Clay B, and (b) Clay C at various plate speeds and on clay that was fresh or used.

The raw data generated by the plate viscometer is available in the repository as a Microsoft Excel file named “Fig. 6a Fig. 7a” for Clay B and “Fig. 6b Fig. 7b” for Clay C. No data was generated for Clay A. Fig. 6 contains an example of the results obtained when subjecting a single sample to measurements in the plate viscometer. For Clay B and Clay C, the maximum viscosity and associated shear rate were derived from the raw data produced. The calculated averages per fixed plate speed were plotted in Fig. 7 for Clay B (Fig. 7(a)) and Clay C (Fig. 7(b)).

## 2 Experimental Design, Materials and Methods

The tap-hole clays were sourced from three different suppliers in South Africa and labelled A, B, and C. Clay A was an experimental clay without datasheet. Clay B and Clay C were available commercially with respective datasheets. Clay B was resin and water-bonded, with mainly silica as aggregate. Clay C was tar and resin bonded, with alumina as the main aggregate. In both instances, the maximum particle size of the aggregate was 3 mm. The WI was not reported for either of the two clays.

**Fig. 8:**
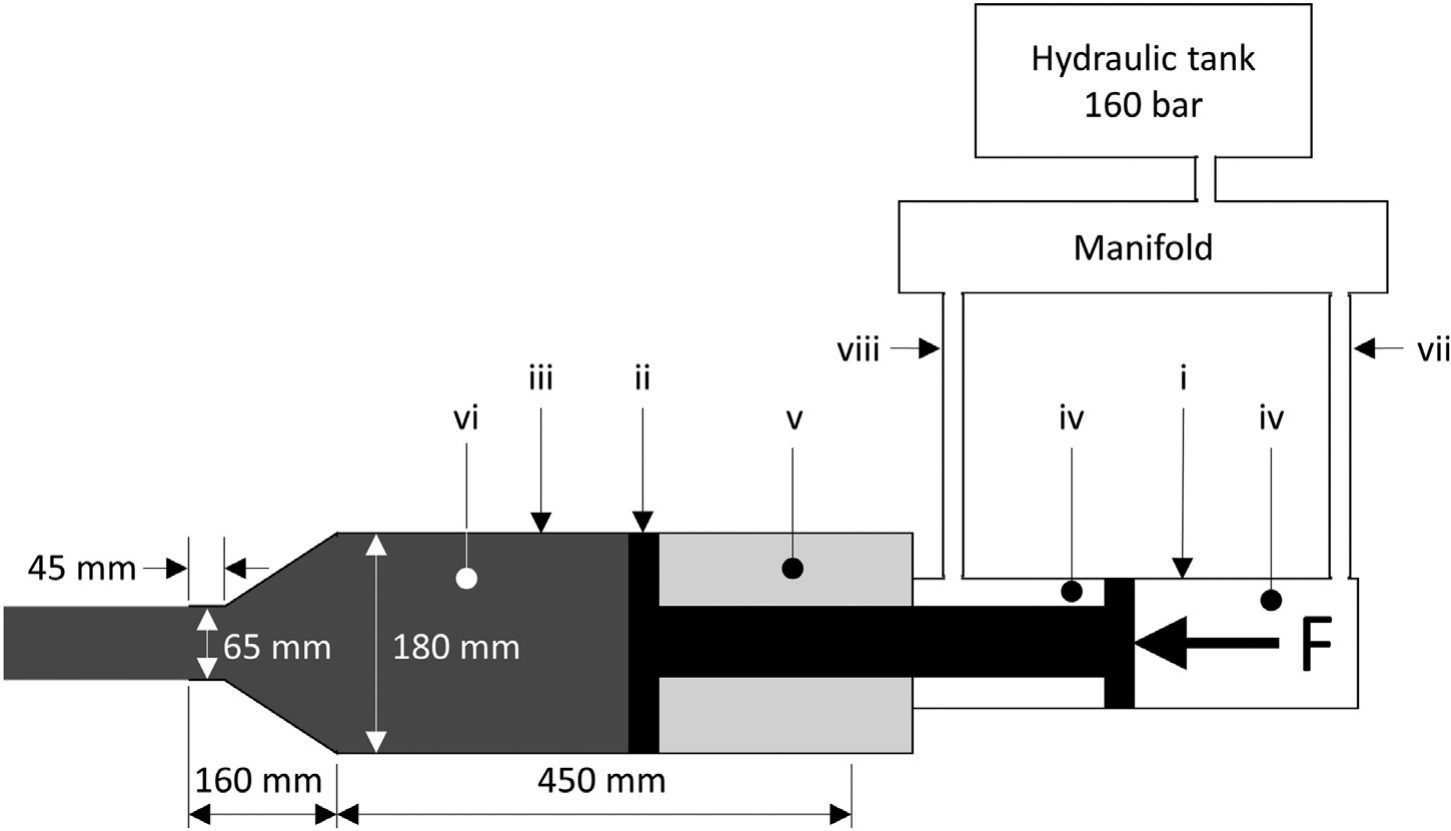
Schematic layout of the pilot-scale clay-gun (reproduced from Steenkamp et al. [1]).

The pilot-scale clay-gun (DDSA/O-M/MTK1160) at Mintek in Johannesburg, South Africa was applied in the testwork. The main parts and dimensions of the clay-gun, applied in the experiments presented here, are illustrated in Fig. 8. The hydraulic cylinder (i) pushes a piston (ii) in the cylindrical barrel (iii), in order to extrude the clay into the tap-hole. The hydraulic cylinder is filled with (iv) oil, and the clay barrel to the back of the piston with (v) air and to the front with (vi) tap-hole clay. When pushing the clay towards the tap-hole, pressurized oil is applied through a pipe (vii) and non-pressurised oil released through another pipe (viii). When the piston is pulled back, in order for the clay to be loaded into the barrel, the process is reversed. P indicates the hydraulic pressure (measured in line vii) applied by the hydraulic system to the clay and is referred to as the ‘plugging pressure’. For this specific design, the clay pressure is one third of the measured plugging pressure due to the clay-gun configuration.

For each experiment, the piston was pulled backed completely and the barrel filled with 11–17 kg of clay, depending on the type of clay. The clay was then extruded fully, as for typical clay-gun operations when conducting a pilot smelting campaign, except that the clay was supported by an angle iron attached to the mouth-piece of the clay-gun during extrusion, rather than being injected into a tap-hole. During the experiment, the plugging pressure was logged automatically. On each clay, at least 5 experiments were conducted and the plugging pressure (measured in the hydraulic system) was logged as a function of time.

**Fig. 9:**
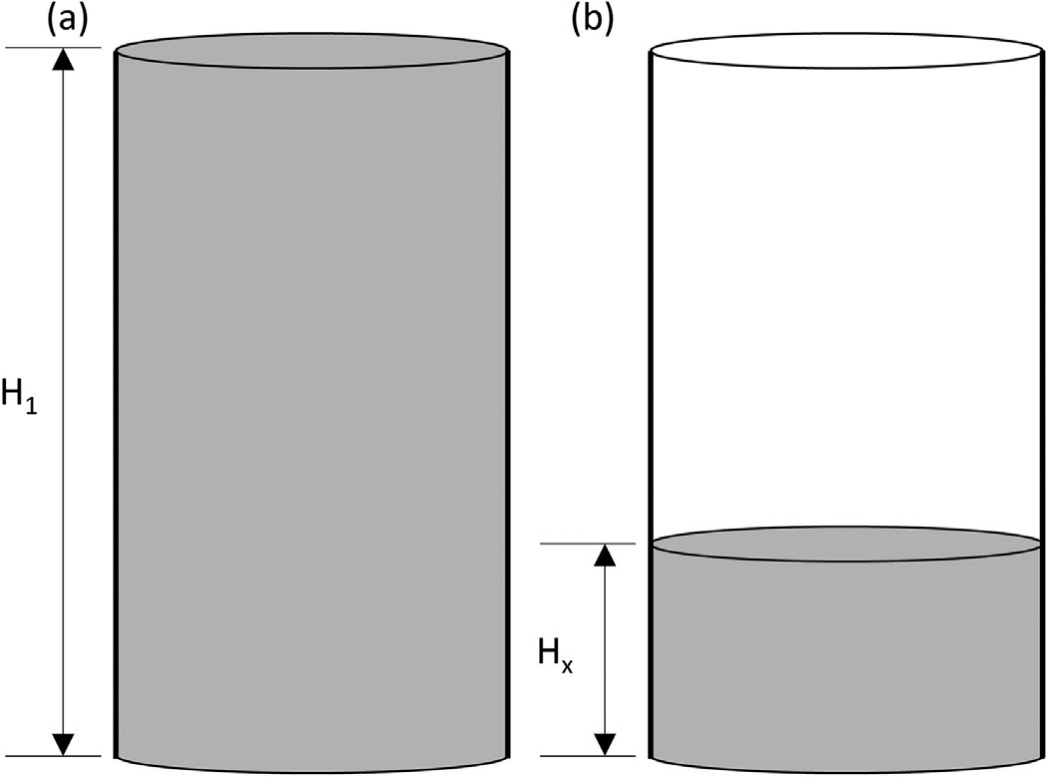
Height after first ram (H_1_) and height after x-number of rams (H_x_) where x represents 4 for Eq. (1) and 100 for Eq. (2).

The automated sand-rammer (R&D Carbon, RDC-194) at Elkem Carbon in Kristiansand, Norway was used to determine the WI and related information. In the first set of experiments, the height of the clay as a function of number of rams were determined for 100 rams per experiment. Five experiments per clay were executed. The experiments were executed according to the methods described in ASTM-C181 [4] and ISO 1927-3 [5]. From these results, the WI (based on the four first rams, calculated in Eq. (1)), the extended WI (based on all 100 rams, calculated in Eq. (2)) and changes in height and changes in density per ram as reported by the automated sand-rammer. The density calculation is based on the weight of the clay, measured prior to the experiments utilizing a laboratory scale, and the height measurements recorded by the sand-rammer. The height parameters utilised in Eqs. (1) and (2) are indicated in Fig. 9.

(1) $$\mathrm{WI}=\frac{100\;\left(H_{1}-H_{4}\right)}{H_{1}}$$

Where:

• WI is the workability index
• H_1_ is the height after the first ram, the first measurement logged by the automated sand-rammer
• H_4_ is the height after the fourth ram
  (2) $$WI_{\mathrm{extended}}=\frac{100\;\left(H_{1}-H_{100}\right)}{H_{1}}$$

Where:

• WI_extended_ is the extended workability index
• H_1_ is the height after the first ram, the first measurement logged by the automated sand-rammer
• H_100_ is the height after the one hundredth ram

The plate viscometer (Viscometer M11) was also located at Elkem Carbon in Kristiansand, Norway [6]. The plate viscometer was built in-house by Elkem to study the viscous behaviour of electrode paste applied in the Ferroalloy and Aluminium industries. Electrode paste is a complex material, similar to tap-hole clay, and typically consists of 70–75% solids and a pitch binder. The pitch binder behaves like a Newtonian fluid as its viscous behaviour is only shear rate dependant, whilst the viscous behaviour of the paste is dependant on both the force applied and the shear rate. Given Elkem's experience with using both sand-rammers (manual and automated) and the plate viscometer in the evaluation of electrode pastes, the option of transferring the technique to the evaluation of tap-hole clays was considered here. The instrument is operated with a constant velocity and is of the constant volume type.

**Fig. 10:**
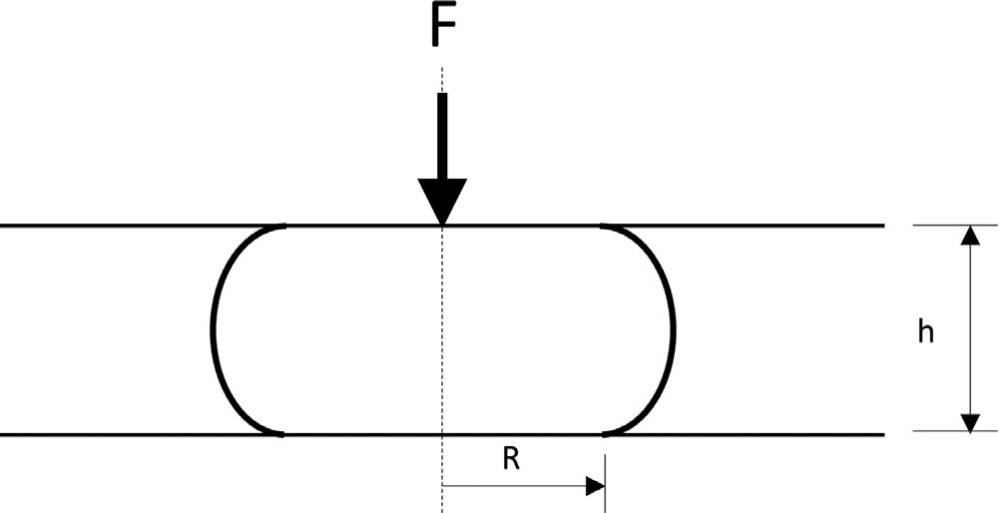
Force (F) and height (h) parameters utilised in Eq. (3) and radius (R) parameter in Eq. (4).

The tap-hole clay sample is prepared and placed on the stationary plate. The sample is then squeezed between two parallel plates, one stationary (on which the sample rests and which measures the force applied) and the other moving. The instrument measures and records the change in height and compression force applied as a function of time, for a fixed plate velocity and sample volume. From the results obtained, the viscosities and shear rates are calculated based on Eqs. (3) and (4) respectively, as published in Tørklep [6]. The force and height parameters utilised in Eq. (3) and radius parameter in Eq. (4) are indicated in Fig. 10.

(3) $$\eta =\frac{2\pi Fh^{5}}{3V^{2}\;v_{p}}$$

Where:

• η is viscosity (kPa.s)
• F the applied force (N)
• h is the distance from the plate (m)
• V is the constant sample volume (m^3^)
• v_p_ is the fixed plate velocity (m/s)
  (4) $$\gamma =-\left(\frac{3R}{h^{2}}\right)\frac{dh}{dt}$$

Where:

• γ is the shear rate (/s)
• R is the sample radius (m)
• $\frac{dh}{dt}$ is the instantaneous plate velocity (m/s)

Between 1 and 4 experiments were conducted per fixed plate speed, and because of the limited availability of the clay, measurements were done on fresh and used clay. No results could be obtained for Clay A as once the clay was removed from the sample holder it flowed under gravity instead of retaining its shape, thus sample preparation at room temperature was not possible. As the flow rates of electrode paste during operation are very low and the instrument was designed for electrode paste, it could only be operated at rates up to 450 mm/min. For Clay B, measurements were made using the plate viscometer at plate speeds fixed at 1, 10, 40, 70, and 450 mm/min. For Clay C, experiments were conducted at plate speeds fixed at 1, 10, 100, 150, 225, and 450 mm/min.

## CRediT Author Statement

**Joalet Steenkamp:** Conceptualization, Resources, Methodology, Investigation, Data Curation, Formal analysis, Visualization, Original Draft, Project administration, Funding acquisition; **Charlotte Lindstad:** Methodology, Investigation, Review & Editing; **Lars Lindstad:** Conceptualization, Review & Editing, Supervision, Funding acquisition.

## Declaration of Competing Interest

The authors declare that they have no known competing financial interests or personal relationships which have or could be perceived to have influenced the work reported in this article.
